# Repeatability of Corticospinal and Spinal Measures during Lengthening and Shortening Contractions in the Human Tibialis Anterior Muscle

**DOI:** 10.1371/journal.pone.0035930

**Published:** 2012-04-26

**Authors:** Jamie Tallent, Stuart Goodall, Tibor Hortobágyi, Alan St Clair Gibson, Duncan N. French, Glyn Howatson

**Affiliations:** 1 School of Life Sciences, Northumbria University, Newcastle-upon-Tyne, United Kingdom; 2 University Medical Center of Groningen, University of Groningen, Groningen, The Netherlands; 3 Centre for Aquatic Research, University of Johannesburg, Gauteng, South Africa; University of Alberta, Canada

## Abstract

Elements of the human central nervous system (CNS) constantly oscillate. In addition, there are also methodological factors and changes in muscle mechanics during dynamic muscle contractions that threaten the stability and consistency of transcranial magnetic stimulation (TMS) and perpherial nerve stimulation (PNS) measures.

**Purpose:**

To determine the repeatability of TMS and PNS measures during lengthening and shortening muscle actions in the intact human tibialis anterior.

**Methods:**

On three consecutive days, 20 males performed lengthening and shortening muscle actions at 15, 25, 50 and 80% of maximal voluntary contraction (MVC). The amplitude of the Motor Evoked Potentials (MEPs) produced by TMS was measured at rest and during muscle contraction at 90° of ankle joint position. MEPs were normalised to Mmax determined with PNS. The corticospinal silent period was recorded at 80% MVC. Hoffman reflex (H-reflex) at 10% isometric and 25% shortening and lengthening MVCs, and V-waves during MVCs were also evoked on each of the three days.

**Results:**

With the exception of MEPs evoked at 80% shortening MVC, all TMS-derived measures showed good reliability (ICC = 0.81–0.94) from days 2 to 3. Confidence intervals (CI, 95%) were lower between days 2 and 3 when compared to days 1 and 2. MEPs significantly increased at rest from days 1 to 2 (P = 0.016) and days 1 to 3 (P = 0.046). The H-reflex during dynamic muscle contraction was reliable across the three days (ICC = 0.76–0.84). V-waves (shortening, ICC = 0.77, lengthening ICC = 0.54) and the H-reflex at 10% isometric MVC (ICC = 0.66) was generally less reliable over the three days.

**Conclusion:**

Although it is well known that measures of the intact human CNS exhibit moment-to-moment fluctuations, careful experimental arrangements make it possible to obtain consistent and repeatable measurements of corticospinal and spinal excitability in the actively lengthening and shortening human TA muscle.

## Introduction

Proposed in 1985 [1] as a non-invasive and pain free method to examine transient functional lesions of the brain [2], transcranial magnetic stimulation (TMS) is a widely used tool to examine motor cortical physiology [2], [3]. Relatively few studies [4], [5], [6], [7] have examined the stability and consistency of TMS measures that provide information on excitability and plasticity of the human nervous system. This is surprising because there are at least two main sources of variation that can affect the stability of TMS measures. One is the constant oscillation in the elements of the human central nervous system (CNS), including the neurons forming the corticospinal tract [8], [9], [10] that contribute to the variable nature of TMS measures. A second source of variation is methodological, in particular, the level of muscle torque and the changing muscle mechanics [5], [10], [11], subject population and the muscle under investigation [5], [6]. To underscore the need for determining the consistency and stability of TMS measures, studies have shown that a few forceful muscle contractions or repetitive actions can readily modulate the excitability of the intact human primary motor cortex (M1) [5], [12], [13]. In addition, many of these TMS protocols were administered over several days but virtually none of these studies report what, if any, effects are due to repeat TMS measurements *per se*. Therefore, it is important to determine the magnitude of day-to-day variation that is due to the administration of the TMS measurements.

The use of TMS in combination with other neurophysiological measures are needed to assess if changes in M1 are mediated at a spinal level [14]. One such measurement that can complement TMS is provided by the peripheral nerve stimulation (PNS) producing the Hoffman reflex (H-reflex), [15], [16]. The H-reflex represents motoneuron excitability and presynaptic inhibition of the motoneuron reflex arc [17], [18], [19]. The reliability of H-reflex is well established at rest in the soleus [20], [21], [22], [23], but less is known about the day-to-day variation in other muscles such as the TA [22], or whilst the muscle changes in length [24]. Compared to shortening and isometric actions, lengthening muscles actions appear to possess unique neurological characteristics in several elements of the CNS between M1 and motor units [25], [26] and it is unclear if these characteristics would affect between-day stability of TMS and PNS measures. Furthermore, TMS or H-reflex alone provides limited information; coupling these techniques in the same exercise paradigm gives further detail of changes in excitability at multiple levels of the central nervous system. To the best of our knowledge, no study has established the repeatability of these methods in a single experiment.

Despite the increasingly amount of experimental studies using TMS and PNS [27], [28], [29], [30] during dynamic actions only a few studies have investigated the repeatability of TMS or PNS in the TA [4], [22], [31]. Surprising there is even less information on the repeatability of these measures during dynamic muscle actions [24], [32]. To date no study has investigated the day-to-day repeatability of TMS and PNS measures in a single trial during dynamic contractions in the TA. A repeatable method to assess cortical and spinal responses from day-to-day may help further understand neurological conditions in the TA. Therefore, the aim of the present study was to assess the day-to-day repeatability of commonly used measures of neuromuscular function and adaptation using both TMS and PNS during lengthening and shortening muscle actions.

## Results

**Figure 1:**
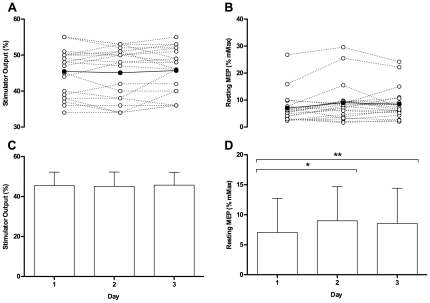
Individual resting motor threshold as a percentage of stimulator output. Clear dots represent individual participants whilst filled dots represent mean data (**A**). Individual and mean resting motor evoked potentials (MEPs) (**B**). Mean resting motor threshold (**C**) and mean resting MEPs as a percentage of Mmax (**D**) on day1, 2, and 3. *(P = 0.016) and **(P = 0.046) denotes significant difference.

**Figure 2:**
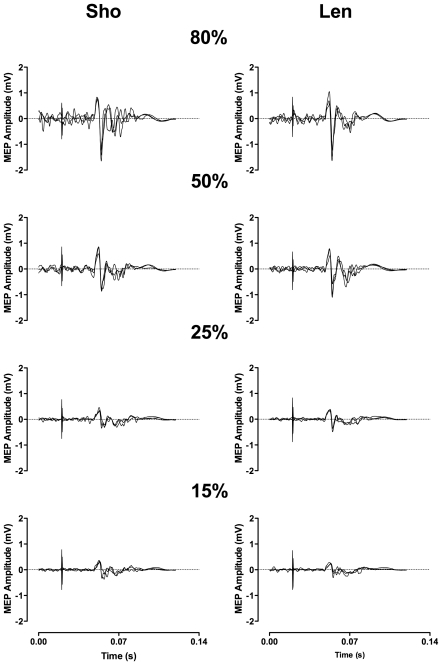
Representative traces of motor evoked potentials overlaid across the three days at 15, 25, 50 and 80% of relative maximal voluntary contractions. **A** = Shortening, **B** = Lengthening.

**Figure 3:**
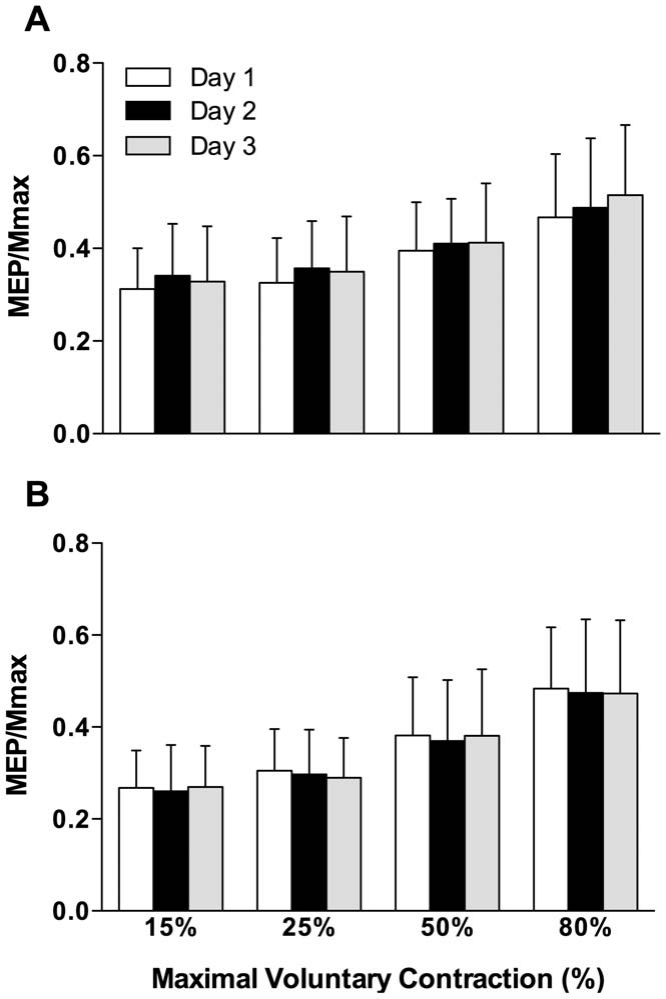
Motor evoked potentials day 1, 2, 3 at 15, 25, 50, and 80% of relative maximal voluntary contraction (MVC). **A** = Shortening, **B** = Lengthening.

**Figure 4:**
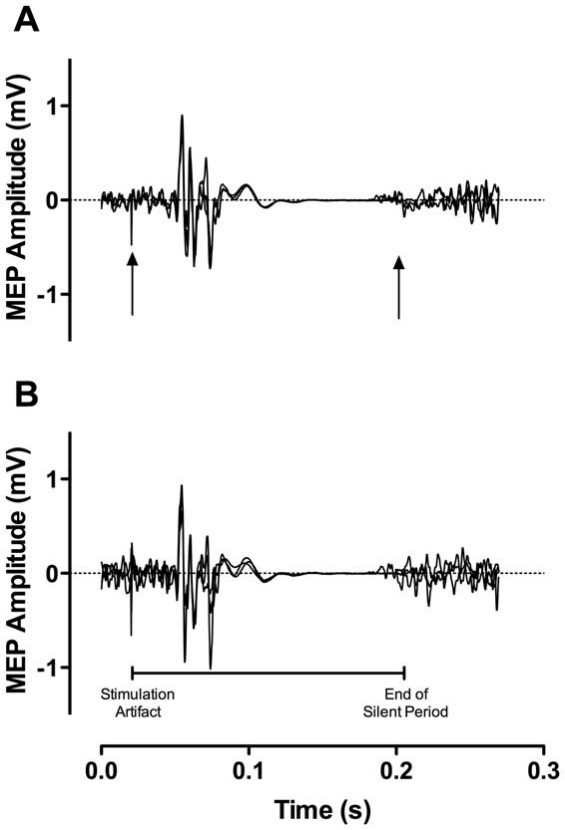
Representative traces of the cortical silent period for shortening (A) and lengthening (B) contractions at 80% of maximal voluntary contraction (MVC) are overlaid across the three days.

A RM ANOVA [contraction (lengthening, shortening) by intensity (15, 25, 50, 80%) by day (1, 2, 3)] showed no significant differences (P>0.05) in relative torque over the 3-day period (Table 1). Therefore, TMS and PNS variables were evoked under the similar contraction intensities between contraction types across the three days. Despite rMT remaining stable, resting MEP was significantly F_(1,19)_ = 4.1; P = 0.025 different between days (Fig. 1). Post-hoc analysis revealed a significant difference in MEP/Mmax between days 1–2 (P = 0.016; 95% CI 0.00 - 0.04) and 1–3 (P = 0.046; 95% CI 0.00 - 0.03) with no difference between days 2–3. A representative trace of the MEPs evoked at different intensities during shortening and lengthening is presented in Fig. 2. Across the three days, there was no change in shortening (P = 0.11) or lengthening (P = 0.14) MEPs (Fig. 3). There was no significant difference in the cortical silent period across the three days (shortening; P = 0.79; lengthening; P = 0.13); a representative trace of the cortical silent period across the 3 days for both contraction types is presented in Fig. 4. No significant differences were reported between days for any PNS variables (Table 2).

**Table 1:** Force (% MVC) of the TA during different shortening and lengthening contraction intensities during TMS and PNS (mean ± SD).

	TMS								PNS	
	SHO	LEN	SHO	LEN	SHO	LEN	SHO	LEN	SHO	LEN
	Target Torque (%)									
	15		25		50		80		25	
**Day 1**	16.9±6.22	19.4±3.95	25.0±4.09	29.9±7.54	48.3±6.71	51.6±7.95	73.1±12.0	77.1±5.85	25.5±7.23	28.1±6.78
**Day 2**	16.1±3.80	19.1±3.26	27.1±5.90	29.1±5.41	50.6±7.81	49.6±7.86	75.9±9.21	75.5±10.66	25.2±4.26	26.8±7.49
**Day 3**	18.0±4.72	18.7±2.95	26.4±4.04	27.7±5.12	48.5±7.26	49.3±8.45	76.0±9.57	73.8±9.08	26.8±4.42	28.0±3.82

TMS, Transcranial Magnetic Stimulation; PNS, Peripheral Nerve Stimulation; ISO, Isometric; SHO, Shortening; LEN, Lengthening.

**Table 2:** Mean ± SD for PNS variables across three consecutive days.

	Mmax	ISO H-reflex	SHO H-reflex	LEN H-reflex	SHO V-wave	LEN V-wave
**Day 1**	4.95±0. 26	12.7±3.5	13.7±6.0	10.6±4.2	44.0±2.0	38.8±1.2
**Day 2**	4.95±0.36	13.0±4.5	13.7±5.9	10.0±3.9	42.1±1.7	35.5±1.3
**Day 3**	5.07±0.30	14.0±5.0	14.4±6.2	9.8±3.9	39.4±1.8	32.6±1.8

Mmax (mV), H-reflex (% Mmax), V-wave (% Mmax).

PNS, Peripheral Nerve Stimulation; ISO, Isometric; SHO, Shortening; LEN, Lengthening.

Excluding those evoked at 80% shortening MVC, the MEPs showed good reliability (ICCs = 0.79–0.92) across the three days (Table 3). The CV and CI were predominantly smaller between days 2–3 when compared to days 1–2 (Table 3). Resting MEPs had the highest overall error (CV = 28.9%) compared to both contraction types and across intensities. Cortical silent period and rMT demonstrated the lowest variability (CV<7.5%) compared to any other cortical response. Reliability varied from moderate to high (ICC = 0.54–0.84) for PNS related variables but showed a predominantly higher CV (11.7–29.3%) than TMS variables. Unlike TMS, there was no apparent familiarisation effect with PNS.

**Table 3:** Coefficient of variation (CV), change in mean confident intervals (CI) and Intraclass correlation coefficients (ICC) across the three days, between days 1 and 2 (D1–D2) and days 2 to 3 (D2–D3) for corticospinal variables.

	ICC			% Change in Mean (95% CI)		CV (%)		
	Overall	D1–D2	D2–D3	D1–D2	D2–D3	Overall	D1–D2	D2–D3
**rMT**	0.93	0.94	0.92	−0.77 (−3.7 −1.8)	1.33 (−1.6 −5.0)	3.2	3.2	3.3
**Rest MEP**	0.87	0.88	0.89	27.7 (−0.2 −54.1)	−5.01 (−15.5 −13.5)	28.9	30.4	15.7
**SHO MEP 15%**	0.83	0.86	0.82	9.15 (−2.0 −17.8)	−3.74 (−15.0 −7.4)	13.2	11.7	13.3
**SHO MEP 25%**	0.92	0.95	0.89	9.74 (4.5 −16.1)	−2.05 (−10.0 −3.3)	9.7	8.8	8.8
**SHO MEP 50%**	0.79	0.73	0.81	3.84 (−5.0 −14.7)	0.58 (−10.4 −8.1)	12.7	11.6	11.3
**SHO MEP 80%**	0.63	0.52	0.73	4.38 (−10.8 −20.5)	5.58 (−4.3 −18.5)	15.4	15.1	12.7
**LEN MEP 15%**	0.88	0.86	0.90	−2.89 (−13.1 −6.4)	3.64 (−5.2 −14.0)	12.1	12.4	10.1
**LEN MEP 25%**	0.88	0.84	0.92	−2.51 (−13.2 −5.7)	−2.38 (−7.3 −4.9)	11.3	11.1	7.4
**LEN MEP 50%**	0.84	0.83	0.85	−3.27 (−14.3 −7.6)	3.00 (−7.3 −14.7)	12.3	12.1	11.7
**LEN MEP 80%**	0.81	0.69	0.92	−1.90 (−14.7 −8.7)	−0.22 (−5.4 −6.9)	13.2	13.9	7.7
**SHO SP**	0.94	0.94	0.94	0.61 (−6.1 −6.2)	0.96 (−2.7 −8.9)	7.4	6.1	5.8
**LEN SP**	0.96	0.98	0.94	3.24 (−0.8 −7.6)	1.66 (−2.2 −8.0)	4.6	6.7	7.1
**Mmax**	0.66	0.72	0.66	−0.14 (−10.3 −8.6)	2.67 (−4.6 −18.0)	11.7	10.9	12.3
**H-reflex**	0.65	0.65	0.66	2.20 (−13.5 −14.2)	7.50 (−7.1 −26.6)	19.1	15.7	17.7
**SHO-H-reflex**	0.84	0.83	0.85	0.28 (−11.6 −15.3)	5.17 (−6.0 −17.4)	15.5	12.2	15.4
**LEN H-reflex**	0.76	0.79	0.74	−6.14 (−16.7 −11.9)	−1.80 (−22.3 −8.8)	16.1	17.2	20.5
**SHO-V-wave**	0.77	0.76	0.76	−4.44 (−16.7 −11.7)	−6.48 (−22.9 −8.6)	22.0	17.3	16.4
**LEN-V-wave**	0.54	0.35	0.63	11.6 (−9.4 −39.8)	8.22 (−30.2 −6.4)	29.3	27.1	25.4

rMT, Resting Motor Threshold; MEP, Motor Evoked Potentials; SHO, Shortening; LEN, Lengthening; SP, Silent Period.

## Discussion

Intrinsic oscillations in the CNS, methodological factors, and muscle mechanics make TMS and PNS measures variable. Here we presented new information focused on the stability of TMS and PNS measures during dynamic muscle contractions. The main finding was that TMS and PNS measures revealed a high degree of repeatability during shortening and lengthening muscle contractions across three consecutive days. Variability in TMS measures, evidenced by lower CV and reduced heteroscedasticity of the 95% CI, decreased from 2^nd^ to 3^rd^ day of testing, therefore a familiarisation session is advisable to improve repeatability. However, this trend is not apparent in PNS measures.

Previous research investigating the reliability of cortical responses in the TA has reported similar ICC values of 0.98 [31] and 0.88 [4] for rMT and resting MEP, respectively. Upper limb muscles have also revealed stable rMT between days [6]; it seems likely that the level of stimulation needed to excite the target muscle remains relatively consistent across repeated days. Despite the high ICC reported for resting MEP, the variability of the resting MEP between day 1–2 was relatively high (CV = 30%). Therefore meaningful detectable changes in cortical excitability would need to be large to detect a worthwhile change. However the variability significantly decreased between days 2 and 3 (CV = 16%), which make the use of a familiarisation session essential. Consistent with previous studies, a single TMS session with multiple contractions can cause changes within M1 [5], [12]. In general, the TA is naturally accustomed to exercises that require smaller forces or resistance; the exposure in this study to higher intensity shortening and lengthening actions was probably unfamiliar for the TA and therefore make the expectation tenable that some degree of plasticity has occurred within the motor cortex. As the mere administration of TMS may also contribute to increased corticospinal excitability, [5] it is likely that both the unaccustomed forceful contractions and TMS stimuli play a role in the increased variability and change in corticospinal excitability from day 1 to 2.

When compared to rest, this study suggests MEPs are more repeatable in an active muscle. With the exception of Kamen (2004), who showed a higher reliability during rest, assessing the motor cortex when the target muscle is activated appears to stabilise MEPs [11], [33]. At rest, sensory inputs may influence the excitability of motor units in the pathway from M1 to the target muscle and thus potentially increase the variability of the MEP [10]. This is further supported with the body of research evidence showing changes in the size of the MEP through mental practice or imagery tasks [34], [35]. Darling *et al.* [10] suggested that the visual display of target torque reduced the variability through channelling the participants' attention to the required task. Although sensory inputs are important, it should be acknowledged that the sub-threshold motoneuron activity, which was not examined, might also influence the MEP response.

Consistent with previous studies, during isometric [5], [11] and dynamic actions [32] we demonstrated a trend toward poorer reliability and highest variability in MEPs at the higher intensities, particularly when the muscle was shortening. The high contraction intensities potentially cause larger desynchronization of the compound action potential at the muscle membrane [11], [36], [37]. The intermittent arrival of the action potential at the muscle disrupts the ‘shape’ of the MEP through phase out cancelation [37]. Furthermore, compared to a lower intensity contraction where torque is achieved through the intermittent activation of numerous motor units, the chance of a TMS pulse being discharged during the neuron refractory period during a high intensity contraction is increased because of greater synchronisation of motor units [10]. Although the results in our study support the work from Darling *et al.* [10], where there was a stabilizing effect of the MEP with a mild muscle contraction, the highest reliability was not at the lowest torque output for shortening or lengthening actions but at an intensity of 25% MVC. This is consistent with previous work showing higher repeatability during active dynamic muscle actions at 20% compared to 10% MVC [32]. The exact reasons for this are unclear but may anecdotally be linked to the participants' motor ability to reach the required level of force at the higher (80%) and lower (15%) intensities during dynamic contractions, which arguably is more challenging.

Compared to previous work during isometric [5] and dynamic contractions [32], we have demonstrated that MEPs can be evoked with low variability between trials. Numerous methodological issues such as the selection of TA as the target muscle, the type of coil and number of stimuli given may account for higher reproducibility reported in our study compared to the previously discussed studies. Interestingly, when compared to lengthening muscle actions, shortening actions showed a poorer reliability at high contraction intensities. A reduced presynaptic synchronisation and a decrease in the probability of extra synchronous discharges during shortening actions [38] could increase the amount of phase out cancelation and thus the variation in MEP amplitude during shortening actions.

The cortical silent period is thought to represent both spinal and intracortical inhibition [39], [40]. One previous study has investigated the reliability of the cortical silent period during dynamic contractions [32] and suggested that the cortical silent period was not repeatable under dynamic muscle actions. However, our results support the data from other work conducted under isometric conditions that the cortical silent period is a stable and repeatable TMS measure from day-to-day [41], [42], [43], [44]. Furthermore, there was no evidence of differences in the repeatability measures between shortening and lengthening muscle actions at 80% MVC. As the cortical silent period is easily defined at high contraction intensities [44] and is not affected by phase out cancelation in the same way as an MEP, it seems that the cortical silent period during 80% shortening and lengthening MVC is highly reliable. Therefore, factors such as contraction intensity [44] and method used to quantify the silent period [41] might have a greater influence on the degree of reliability.

H-reflex is a reliable and well established method to assess spinal excitability at rest [20], [22] and during isometric contractions [45]. Our results add to the limited research conducted during dynamic conditions [24] and showed only a small increase in variability when H-reflex is evoked during a dynamic contraction. Many studies examining muscles of the leg have predominantly focussed on the soleus and gastrocnemius rather than TA, using PNS techniques; perhaps because of the ease to stimulate the tibial versus peroneal nerve. However, differences in the neuromechanics of muscle recruitment may also play an important role in the choice of muscle and therefore repeatability of the H-reflex. For example, the EMG response from transcutaneous stimulation of dorsal roots within the lumbosacral cord is higher in the soleus when compared to the TA [46], [47]. Therefore, despite no differences in the site of stimulation there is an apparent difference in recruitment strategies of the muscle that may contribute to the reduced repeatability of the TA when compared to the soleus. An additional possibility for the higher variability of H-reflex in the TA may reside with M_MAX_. Although there was no significant difference in M_MAX_, and a high degree of repeatability was also found (ICC = 0.66–0.72), the between trial ICC reported in previous work examining soleus and flexor carpi radialis was moderately higher (ICC≥0.75) [45], [48], [49]. This may account for the greater variability in H-reflex; however, interestingly MEPs were also normalised to M_MAX_ and showed a very high degree of repeatability and therefore suggests that H-reflex itself is a more variable measure from day-to-day in the TA.

The V-wave is often used as a measure of corticospinal drive [18], [50], [51]. Only one study has investigated its day-to-day reliability [52]. The authors in that study showed that V-waves evoked during an isometric contraction of the gastrocnemius and soleus can be reliable from day-to-day (ICC = 0.92 and 0.86, respectively). Our results support this finding during shortening muscle actions (ICC = 0.77), and to a lesser extent during lengthening actions (ICC = 0.54). Notwithstanding the limitations of surface EMG [53], V-wave is somewhat reliant on the antidromic action potential from the electrical stimulation that collides with the voluntary drive, but can also be influenced by motoneuron excitability and pre- and post-synaptic inhibition [52]. Speculatively, the dynamic contractions used in our investigation may show a small, but nonetheless a greater degree of variability in the collision or excitability of the motoneuron, although future research is required to elucidate underlying mechanisms of V-wave [52], particularly during different muscle actions.

In summary, although variation in intrinsic and methodological sources of error present a threat to the stability of TMS and PNS measures of excitability, we have demonstrated that such measures are consistent and stable in the TA across three consecutive days. The data suggest greater repeatability and lower scedasticity from day 2 to day 3 than day 1 to day 2, therefore it seems prudent to include a familiarization session to reduce the error associated with TMS measures in the TA, but this does not seem necessary for PNS measures.

## Materials and Methods

### Participants

Prior to the start of the investigation, ethical approval was gained from Northumbria University Ethics Committee in accordance with the Declaration of Helsinki. Twenty healthy males volunteered to take part in the study (age 24±3 yrs, 177±7 cm, 82±3 kg). All participants were screened for neurological disorders, pacemakers and intracranial plates [54] and provided written informed consent. The dominant leg was determined using a previous method [55], which included asking participants to stamp the ground, kick a soccer ball and push an object with their foot. Of the 20 participants, 18 were right and 2 were left leg dominant.

### Experiment Design

Participants reported to the laboratory on 3 consecutive days for up to 120 min at the same time of day to avoid diurnal variation. Contraction type (lengthening and shortening), intensity (80, 50, 25 and 15% MVC) and the order of TMS and PNS were pseudo-randomised for each participant. The order was kept consistent for each participant on days 1, 2 and 3. The participants were instructed to arrive at the laboratory in a rested and fully hydrated state. They were also asked to refrain from caffeine and alcohol for 12 and 24 h before each test, respectively.

### Experimental Set-up

Participants were seated in an isokinetic dynamometer (Cybex Norm, Cybex International, NY) with the hip, knee and ankle of the dominant leg set at joint angles of 90, 120 and 90°, respectively, as recommended by manufacturers guidelines. The foot of the dominant leg was firmly strapped into the ankle adapter of the dynamometer whilst the knee was secured in a thigh stabiliser to prevent any extraneous movement of the upper leg. Participants performed dorsiflexion by resisting or assisting (dependent upon contraction type) as the dynamometer moved through 30° of dorsi- and plantar-flexion. Torque feedback was displayed on the monitor of the dynamometer approximately 1 m from the participant.

### Maximal Voluntary Contraction

At the beginning of the initial testing session shortening, lengthening and isometric maximal voluntary contractions (MVC) of the TA were recorded. From a starting position of 75° for lengthening and 105° for shortening contractions, MVCs were recorded as the ankle passed anatomical zero (90°) at a set speed of 15°/s (2 s contraction). During each during each MVC participants were instructed to focus on solely activating their TA. The highest value from 3 trials was recorded as the MVC. From the maximal values, 80, 50, 25 and 15% of shortening and lengthening MVC were calculated. Participants also performed an isometric MVC with the ankle set at 90°. An isometric contraction of 10–15% MVC was used to stabilise the H-reflex to maximal M-wave (M_MAX_) curve (H-M).

### Electromyography

Surface Electromyography (EMG) was recorded over the TA and the lateral gastrocnemius using pairs of electrodes (22 mm diameter, model; Kendall, Tyco Healthcare Group, Mansfield, MA, USA) spaced 2 cm apart. For the TA, electrodes were placed at one-third distance of the line between the tip of the fibula and the tip of the medial malleolus [56]. Electrodes for the lateral gastrocnemius were place at one-third distance of the line between the head of the fibula and the calcaneus. The reference electrode was placed over the medial malleolus. All sites were shaved, abraded with preparation gel and then wiped clean with an alcohol swab. Each site was marked with semi-permanent ink to ensure a consistent placement over the three trials. EMG was amplified (1000×), band pass filtered 10–1,000 Hz (D360, Digitimer, Hertfordshire, UK) and sampled at 5,000 Hz (CED Power 1401, Cambridge Electronics Design, Cambridge, UK).

### TMS Protocol

Motor evoked potentials were elicited via stimulation on the contralateral hemisphere of the dominant leg using a magnetic stimulator (Magstim 200^2^, Magstim Company Ltd, Whitland, UK), with a concave double-coned 110 mm coil (maximal output of ∼1.4 T). The ‘hotspot’ or optimal site for activation of the TA, has previously been reported [33] to be 0.5–1 cm posterior and along the anteroposterior plane of the vertex, thus searching began here. The coil was positioned to induce a posterior to anterior current in the underlying motor cortex. Once optimal coil placement was established, the position was marked directly on the scalp with a permanent marker to ensure consistent placement over the three trials. Resting motor threshold (rMT) was determined as the lowest stimulator output needed to evoke a peak-to-peak MEP greater ≥50 µV in 5 out of 10 consecutive pulses [57]. The rMT was recorded as a percentage of maximal stimulator output. All subsequent MEPs at rest and during contraction were delivered at a stimulator output equivalent to 120% rMT and were averaged over eight stimuli. The MEPs were reported relative to the highest M-wave (M_MAX_) during the H-M recruitment curve (see Peripheral Electrical Stimulation Procedure). The same investigator was used for all trials and all participants.

In a randomised but counterbalanced order, participants performed shortening or lengthening contractions at 80, 50, 25 and 15% MVC. All contractions were separated by at least 25 s [58]. Clear instructions were given to reach the target force as quickly as possible and maintain the required force throughout the duration of the contraction. Before any TMS pulse was delivered during an active contraction, all participants practiced until they were competent at achieving the required force, which generally took 2 or 3 attempts. Participants were exposed to a minimum of 110 TMS stimuli and ∼20 additional stimuli to map the hotspot.

### Peripheral Electrical Stimulation Procedure

Electrical stimulation was administrated below the head of the fibula, on the peroneal nerve using a 40 mm diameter cathode/anode arrangement (pulse 1 ms; Digitimer DS7AH, Welwyn Garden City, Hertfordshire, UK). To ensure a stable H-reflex, each participant was instructed to hold an isometric dorsiflexion contraction of 10–15% MVC. Once the optimal site of stimulation was established, the site was marked with semi-permanent ink and the stimulator strapped to the participant's leg. The H-M recruitment curve consisted of a minimum of 64 pulses below the first appearance of H-reflex and M_MAX_. The max H-reflex was defined as the average of the three highest responses [59].

Following the H-M recruitment curve participants performed 12 shortening and 12 lengthening contractions at 25% MVC; each contraction was separated by 60 s. Low contraction intensity was used to ensure the H-reflex in the TA was easily identifiable with background electromyography. Similarly to others [60], [61], the stimulator output was manipulated to elicit an H-reflex with M-wave amplitude of 15–25% of M_MAX_. Contractions that did not meet this criterion were rejected. As the amplitude of M_MAX_ is affected by intensity of contractions [62], the first two of the 12 lengthening and shortening muscle actions were to determine individual intensity specific M_MAX_ amplitudes. If M-wave did not fit the criteria (15–25% M_MAX_) the H-reflex was excluded. It took the examiner between 2–4 contractions to achieve the appropriate stimulator intensity. Participants were passively moved into position 10 s before performing a submaximal contraction targeted at 10–15% MVC to prevent any thixotropic effect [63]. Finally participants' V-wave was examined with four maximal shortening and lengthening contractions with a supramaximal stimulus 150% of M_MAX_ [18]. V-wave was normalised to resting M_MAX_ from the H-M recruitment curve.

### Data Analysis

Electromyography was recorded 50 ms prior to magnetic stimulation and 500 ms post. The MEPs, cortical silent period and torque were all analysed post trials (Signal 3.0, Cambridge Electronics, Cambridge, UK). The MEP amplitudes were normalised to peak-to-peak M_MAX_. Previous research has shown mathematical modelling of the silent period to be extremely reproducible [41]. Therefore the cortical silent period was measured as the distance from the stimulation artefact to a return of 1 SD of pre-stimulus EMG of pre-stimulus levels.

### Statistics

Data is presented as mean ± standard deviation (SD). To detect significant differences in all parameters (apart from MEP and torque) between days, a one way repeated measures ANOVA was conducted. Two-way repeated measures ANOVA on day (1, 2 and 3) and contraction intensity (80, 50, 25 and 15%) was used to examine differences for lengthening and shortening MEPs. Three-way repeated measures ANOVA on day, contraction type (shortening and lengthening) and contraction intensity was used to test for within group differences in torque. If significant interactions were revealed, LSD *post-hoc* analysis was used for pairwise comparisons. Between-day reliability for each of the variables was assessed by intraclass correlations coefficient (ICC) from days 1–2, 2–3 and across the three days. Additionally, 95% confidence intervals (CI) were determined to assess the magnitude of change and the coefficient of variation (CV) was determined to assess the reliability between days. Statistical analyses were performed using SPSS (version 17.0, Chicago, Illinois, USA).
